# Effects of In-Person Navigation to Address Family Social Needs on Child Health Care Utilization: A Randomized Clinical Trial

**DOI:** 10.1001/jamanetworkopen.2020.6445

**Published:** 2020-06-01

**Authors:** Matthew S. Pantell, Danielle Hessler, Dayna Long, Maoya Alqassari, Christine Schudel, Ellen Laves, Denisse E. Velazquez, Anais Amaya, Patricia Sweeney, Abigail Burns, Francis L. Harrison, Nancy E. Adler, Laura M. Gottlieb

**Affiliations:** 1 Department of Pediatrics, University of California, San Francisco; 2 Center for Health and Community, University of California, San Francisco; 3 Department of Family and Community Medicine, University of California, San Francisco; 4 Social Interventions Research and Evaluation Network, Department of Family and Community Medicine, University of California, San Francisco; 5 Center for Child and Community Health, UCSF Benioff Children’s Hospital Oakland, Oakland, California; 6 UCSF Benioff Children’s Hospital Oakland, Oakland, California; 7 now with Latino Community Foundation, San Francisco, California; 8 School of Nursing, University of California, San Francisco; 9 now with Planned Parenthood Mar Monte, San Jose, California; 10 Department of Obstetrics and Gynecology, Brigham and Women’s Hospital, Boston, Massachusetts; 11 School of Medicine, University of California, San Francisco; 12 Department of Psychiatry, University of California, San Francisco

## Abstract

**Importance:**

While many organizations endorse screening for social risk factors in clinical settings, few studies have examined the health and utilization effects of interventions to address social needs.

**Objective:**

To compare the acute care utilization effects of a written resources handout vs an in-person navigation service intervention to address social needs.

**Design, Settings, and Participants:**

In this secondary analysis of a randomized clinical trial, 1809 adult caregivers of pediatric patients seen in primary and urgent care clinics of 2 safety-net hospitals in northern California were recruited between October 13, 2013, and August 27, 2015. Each participating family was randomly assigned to an in-person navigator intervention vs active control to address the family’s social needs. Analyses were conducted between February 28, 2018, and September 25, 2019.

**Interventions:**

Caregivers either received written information about relevant local resources related to social needs (active control) or met with a patient navigator focused on helping them resolve social needs (navigator intervention). After an initial in-person visit, navigation services included telephone, email, and/or in-person follow-up for up to 3 months.

**Main Outcome and Measures:**

Child emergency department visit or hospitalization within 12 months of study enrollment.

**Results:**

Among the 1300 caregivers enrolled in the study without missing follow-up data, most spoke English (878 [67.5%]) and were women (1127 [86.7%]), with a mean (SD) age of 33.0 (9.33) years. Most children were aged 0 to 5 years (779 of 1300 [59.9%]), 723 children (55.6%) had Hispanic ethnicity, and 462 children (35.5%) were in excellent health; 840 families (64.6%) were recruited from urgent care. In total, 637 families (49.0%) were randomized to the in-person navigator group and 663 (51.0%) to the active control group. There was no difference in risk of an emergency department visit between the 2 groups. Children enrolled in the in-person navigator group had a decreased risk of hospitalization within 12 months (hazard ratio, 0.59; 05% CI, 0.38-0.94; *P* = .03), making them 69% less likely to be hospitalized.

**Conclusions and Relevance:**

In this randomized clinical trial evaluating heath care utilization effects of programs designed to address social needs among families, children enrolled in the navigation group were significantly less likely to be hospitalized after the intervention but equally likely to have an emergency department visit. These findings strengthen our understanding of the effects of addressing social needs in clinical settings as part of a comprehensive strategy to improve health and reduce health care utilization.

**Trial Registration:**

ClinicalTrials.gov Identifier: NCT01939704

## Key Points

**Question:**

Can an in-person service navigation intervention to address family social needs decrease child health care utilization?

**Findings:**

In this randomized clinical trial of 1300 families, provision of an in-person resource navigator significantly decreased the risk of child hospitalization during a 1-year period compared with written information.

**Meaning:**

These findings suggest that providing a patient navigator to address family social needs can decrease child health care utilization.

## Teaser

This secondary analysis of a randomized clinical trial compares the effect on pediatric acute care utilization of a written resources handout vs an in-person navigation service intervention to address family social needs.

## Introduction

A large and compelling body of evidence links social risk factors and child health outcomes.^1,2^ Recently, medical professional organizations such as the American Academy of Pediatrics,^3^ the American Association of Family Physicians,^4^ and the National Academy of Medicine^5^ have endorsed screening for social risk factors in clinical settings. To date, uptake of screening practices has been inconsistent, although more likely in settings serving high numbers of low-income patients.^6,7^

Despite general enthusiasm for social risk screening, few studies have documented the health effects of interventions designed to reduce identified social needs. To date, most studies on social care interventions in clinical settings have described process and social risk outcomes rather than outcomes on child health or health care utilization.^1,8^ When health and/or utilization outcomes have been included in pediatrics-based studies, effects have been inconsistent. Only some studies report child health improvements (eg, improvements in asthma severity scores)^9,10,11^; fewer have demonstrated reductions in avoidable utilization (eg, acute care or emergency department [ED] visits).^11,12^

In this study, we contribute to this rapidly evolving literature by investigating the effect on acute health care utilization in the 12 months following enrollment in 1 of 2 social care interventions designed to assist caregivers of pediatric patients with access to social resources. We hypothesized that the intervention providing an in-person patient care navigator would reduce acute health care utilization in the 12 months following enrollment compared with the intervention providing written resources.

## Methods

### Setting, Participants, and Eligibility Criteria

We conducted a secondary analysis of child health care utilization data collected 12 months after enrollment in a multisite randomized clinical trial. Study methods and results on primary outcomes have been published previously.^9,13^ In brief, caregivers of children being seen in primary and urgent care clinics were recruited from 2 safety-net health systems in northern California between October 13, 2013, and August 27, 2015. Caregivers were eligible for participation if they were aged 18 years or older, spoke English or Spanish, were knowledgeable about the child’s household social characteristics, and lived in the county in which study recruitment took place. Caregivers of children with severe illnesses were excluded. Only 1 child and caregiver per household were enrolled. Navigators were student volunteers from surrounding universities who received 8 hours of training on study recruitment procedures in addition to training to be community service navigators, which involved learning about local social resources and additional skills training in cultural humility and motivational interviewing. Study recruitment and intervention follow-up activities were conducted between October 13, 2013, and August 27, 2015. This study was approved by the Children’s Hospital and Research Center Oakland institutional review board and the Committee on Human Research of the University of California, San Francisco. This study followed the Consolidated Standards of Reporting Trials (CONSORT) reporting guideline.

### Study Design

Due to ethical concerns about a true control group, among whom nothing would be done after screening and identifying unmet social needs, we randomized caregiver-child dyads in 1 of 2 interventions: a written resources intervention (active control) group and a navigation intervention group. We obtained written informed consent for analysis of survey data from all participants at the enrollment visit. Analyzing utilization data was added as an institutional review board modification in the middle of the study; thus, those recruited before this modification were sent a letter asking for consent for electronic health record (EHR) data to sign and return, and those recruited after consented at enrollment.

### Written Resources

Caregivers endorsing any social risks who were randomized to the written resource group were provided preprinted local community resource guides about available county social services, which were not tailored to the families’ endorsed needs. Families in the written resource group did not receive in-person patient navigator assistance on the day of recruitment or after.

### Patient Navigator

In a second group, caregivers endorsing any social risks were invited to meet with a patient navigator, either in person following the clinic visit or by telephone or email if the family had to leave immediately after the visit. Using protocolized social resource algorithms, navigators contacted families every 2 weeks via telephone, email, or in person for up to 3 months or until either identified needs were met or caregivers declined ongoing assistance. Navigators provided assistance connecting caregivers with clinic, government, or community resources targeted specifically to the social barriers that had been endorsed and prioritized by the caregiver. Algorithms can be found online.^14^

### Study Procedures

Computer-assisted randomization was used to assign specific clinic days as navigation intervention days vs written resource days, with day as the unit of randomization and month as the block of randomization. Navigators were unmasked to the group because of the nature of the interventions. They approached eligible families between 9:00 am and 8:30 pm. Families were told they could decline. If they consented to participate, navigators then administered a baseline survey asking about sociodemographic characteristics of the patients and their families and about family social risk factors. These questions were asked to families in both the navigator intervention and active control group. Navigators then provided written resources only on active control days and in-person navigation services on intervention days. The survey took roughly 10 minutes to complete. Caregivers who identified acute caregiver or child mental health or other needs or child abuse in the course of completing study surveys were referred to an onsite social worker or other behavioral health professional, regardless of treatment team. The trial protocol can be found in Supplement 1.

### Measures

#### Demographic Characteristics

The baseline survey administered on the enrollment date included questions regarding child and caregiver age, child and caregiver sex, child race/ethnicity, baseline child health, whether the caregiver had been asked about nonmedical needs in the past 12 months in a clinical setting, and family income based on percentage of the federal poverty level. Because of the nonnormal distribution of child age, we divided age into the 3 following categories: 0 to 5, 6 to 12, and 13 to 18 years. Because of the small number of caregivers (17 of 1300 [1.3%]) reporting their child as having poor health, we combined fair and poor baseline health into 1 category (vs excellent, very good, and good).

#### Social Risks

Baseline household social risk data were collected using a questionnaire described in previous reports.^9,15^ It asked about the following social domains: food insecurity, problems paying utility bills, problems finding employment, housing instability, living in an unhealthy environment, other housing concerns, problems paying medical bills, lack of health insurance, being cut off or denied access to programs that provide income support, lacking a primary care physician, disability impairing ability to work, lack of access to mental health care for someone in the household, problems with a current or former job, and concerns about pregnancy-related work benefits.

#### Health Care Utilization

Data on the number and date of ED visits during the 12 months following the date of enrollment were abstracted from EHRs at both hospitals for all families that provided written consent. The same procedure was followed for hospitalizations.

### Sample Size

The analytic sample comprised 1300 caregiver participants. This allowed us to detect a difference in hazard ratios of 0.85 for ED visits and 0.72 for hospitalizations using 80% power in 2-sided tests with a type I error of 5%.

### Statistical Analysis

The original trial protocol specified a 4-group study based on recruitment setting and intervention, as follows: navigator intervention group recruited from primary care; navigator intervention group recruited from urgent care; active control group recruited from primary care; and active control group recruited from urgent care. However, given that there were no significant interactions between setting, intervention, and utilization outcomes (data not shown), we collapsed by setting, combining the primary and urgent care groups.

We calculated percentages of the sample by sociodemographic factors and used *t* tests and χ^2^ tests to compare characteristics of those included in the analytic sample with those excluded. We used χ^2^ tests to compare the overall rates of having at least 1 ED visit and at least 1 hospitalization during the 12-month postenrollment period. We also constructed Kaplan-Meier survival tables and computed log-rank statistics to assess time to ED visit and time to hospitalization by intervention group. We assessed risk of utilization using Cox proportional hazard regressions without censoring. The χ^2^ testing allowed us to examine whether there were differences in overall outcome rates by intervention group; the Cox proportional hazard regressions enabled us to assess whether there were differences in the time to event occurrence by comparing instantaneous probabilities of events.

For both outcomes, we calculated 3 Cox regression models, as follows: unadjusted; adjusting for site, enrollment setting, child age, child sex, child race/ethnicity, caregiver sex, caregiver age, baseline child health, baseline number of social risks, and whether the caregiver was asked about nonmedical needs in a clinical setting in the 12 months before the visit; and additionally adjusting for poverty, reflecting a smaller sample size. To ensure our models met the assumption of proportional hazards, we performed several analyses including a proportional-hazards assumption test on the basis of Schoenfeld residuals, log-log plots of survival, and including time dependent covariates in the models, which revealed no concerns of our intervention variable violating this assumption.

While the original trial had specified assessing health via a survey at 6 weeks and 4 months after the intervention, this was collapsed to 4 months because of participant burden. However, because we consented families to analyze data through 12 months after enrollment, we include analyses through 4 months of follow-up as a supplement. As a sensitivity analysis, we performed the same series of Cox regression models predicting health care utilization using multiple imputation to account for missing data.

A cutoff value of *P* < .05 was used to determine statistical significance, and all tests were 2-tailed. All analyses were performed using Stata version 15.0 (StataCorp). Analyses were conducted between February 28, 2018, and September 25, 2019.

## Results

### Sample Characteristics

**Figure 1.**
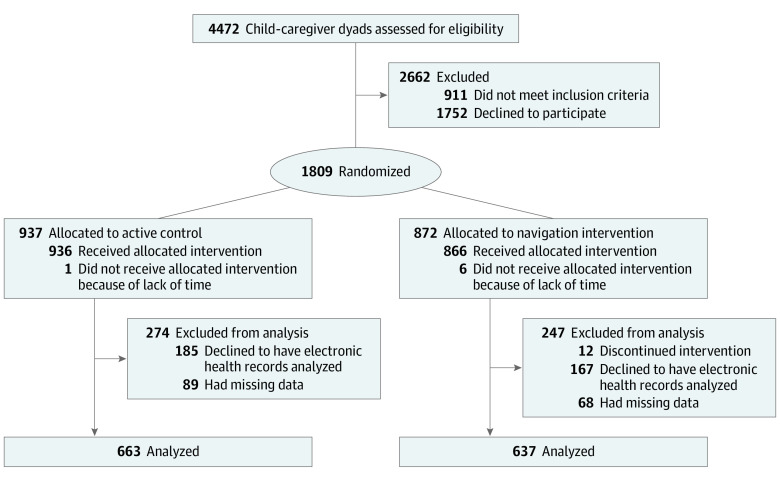
CONSORT Diagram

Of 4472 caregivers invited to participate, 1809 (40.5%) agreed, and 937 (51.8%) were randomized to the active control group vs 872 (48.2%) to the navigator intervention group. Of the randomized participants, 509 either did not consent to having their children’s EHR data analyzed or had missing data, leaving a final analytic sample for this study of 1300 (71.9%), with 637 families (49.0%) in the in-person navigator group and 663 (51.0%) in the active control group. Of these, 184 families (14.2%) declined to provide data about income (Figure 1). Most children were aged 0 to 5 years (779 [59.9%]), 723 (55.6%) had Hispanic ethnicity, and 840 (64.6%) were recruited from urgent care. Most caregivers (878 [67.5%]) spoke English. Approximately one-third of children in each group had excellent health at baseline (intervention, 257 [38.8%]; control, 205 [32.2%]) (Table 1).

**Table 1.** Characteristics by Intervention Group

Characteristic	Intervention group, No. (%)	
	Written resources (n = 663)	Patient navigator (n = 637)
Site		
UCSF Benioff Children's Hospital Oakland	330 (49.8)	321 (50.4)
Zuckerberg San Francisco General Hospital and Trauma Center	333 (50.2)	316 (49.6)
Setting		
Urgent care	432 (65.2)	408 (64.1)
Primary care	231 (34.8)	229 (36.0)
Child age, y		
0-5	385 (58.1)	394 (61.9)
6-12	206 (31.1)	184 (28.9)
13-18	72 (10.9)	59 (9.3)
Female children	353 (53.2)	299 (46.9)
Race/ethnicity		
Hispanic	378 (57.0)	345 (54.2)
Non-Hispanic black	180 (27.2)	178 (27.9)
Asian	32 (4.8)	34 (5.3)
Non-Hispanic white	27 (4.1)	27 (4.2)
Other^a^	46 (6.9)	53 (8.3)
Caregiver language		
Spanish	216 (32.6)	206 (32.3)
English	447 (67.4)	431 (67.7)
Caregiver age, mean (SD), y	33.2 (9.3)	32.9 (9.3)
Women caregivers	582 (87.8)	545 (85.6)
Caregiver relationship to child, No./total No. (%)		
Parent	637/662 (96.2)	617/636 (97.0)
Legal foster parent or guardian	1/662 (0.2)	3/636 (0.5)
Other adult family member	24/662 (3.6)	16/636 (2.5)
Caregiver education		
<8th grade	106/658 (16.1)	110/630 (17.5)
Some high school	116/658 (17.6)	109/630 (17.3)
High school graduate or GED	164/658 (24.9)	178/630 (28.3)
Some college	163/658 (24.8)	154/630 (24.4)
College graduate	109/658 (16.6)	79/630 (12.5)
Social needs, mean (SD), No.	2.6 (2.0)	2.9 (2.2)
Child baseline health status		
Fair or poor	60 (9.1)	58 (9.1)
Good	182 (27.5)	200 (31.4)
Very good	164 (24.7)	174 (27.3)
Excellent	257 (38.8)	205 (32.2)
Asked about nonmedical needs in past year	98 (14.8)	121 (19.0)
Below federal poverty level, No./total No. (%)	410/571 (71.8)	410/545 (75.2)

Abbreviation: UCSF, University of California, San Francisco.

^a^ Other included those selecting from the following: Native Hawaiian or Pacific Islander, American Indian or Alaska Native, and other or mixed race.

However, there were a few differences between the 2 groups. Compared with the active control group, the navigator intervention group had fewer female children (299 [46.9%] vs 353 [53.2%]), more families that endorsed being asked about nonmedical needs in the last year (121 [19.0%] vs 98 [14.8%]), and a higher mean (SD) number of social risks endorsed by families (2.9 [2.2] vs 2.6 [2.0]), justifying our inclusion of these variables as covariates (Table 1).

Compared with patients excluded because of missing data, those included in the analytic sample had higher rates of being recruited from primary care (112 [22.0%] vs 460 [35.4%]; *P* < .001), speaking English (284 [55.8%] vs 878 [67.5%]; *P* < .001), and having fair or poor health (30 of 506 [5.9%] vs 118 [9.1%]) or having excellent health (166 of 506 [32.8%] vs 462 [35.5%]) (*P* = .03) (eTable 1 in Supplement 2). Among families in the full sample navigator intervention group with social needs identified, caregivers participated in a mean (SD) number of follow-up meetings of 1.4 (1.6), with a range of 0 to 13 during the 3 months after enrollment.

### Acute Care Utilization by Navigation Intervention and Active Control Groups

The percentage of children with at least 1 ED visit within the 12-month postenrollment period did not significantly differ by intervention group, with 236 children (37.1%) in the intervention group vs 250 children (37.7%) in the active control group, which corresponded to risk difference between navigator intervention vs active control group of −0.7% (95% CI −5.9% to 4.6%), and a relative risk of 0.98 (95% CI, 0.85 to 1.13). However, significantly fewer children from the in-person navigation group (29 [4.6%]) were admitted to the hospital during the year following enrollment compared with children from the active control group (50 [7.5%]; risk difference, −3.0%; 95% CI, −5.6% to −0.4%; relative risk, 0.60; 95% CI, 0.39 to 0.94). In total (including multiple ED visits and multiple hospitalizations), the active control group had 414 ED visits, with a mean of 0.62 ED visits per child (SD, 1.08; 95% CI, 0.54 to 0.71), as well as 55 hospitalizations, with a mean of 0.08 hospitalizations per child (SD, 0.30; 95% CI, 0.06 to 0.11). The patient navigator intervention group had a total of 404 ED visits and 35 hospitalizations, averaging 0.63 ED visits (SD, 1.04; 95% CI, 0.55 to 0.72) and 0.05 hospitalizations (SD, 0.30; 95% CI, 0.03 to 0.08) per child.

**Figure 2.**
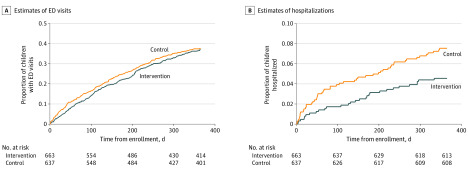
Kaplan-Meier Survival Estimates of Emergency Department (ED) Visits and Hospitalizations

Kaplan-Meier curves revealed no statistically significant difference between time to ED visit within 1 year of enrollment (log-rank *P* = .66). We found significant differences by intervention group in time to hospitalization (log-rank *P* = .02) (Figure 2).

Using Cox proportional hazard regression, we found that during the study follow-up period, children enrolled in the navigator intervention group had a decreased risk of being hospitalized (hazard ratio, 0.59; 95% CI, 0.38-0.94; *P* = .03), making them 69% less likely to be hospitalized than children in the active control group. There was \no change in risk of having an ED visit (hazard ratio, 0.96; 95% CI, 0.80-1.14; *P* = .81). The former finding remained significant when controlling for all sociodemographic variables (hazard ratio, 0.59; 95% CI, 0.35-0.99; *P* = .046) (Table 2). The patient navigator intervention effects on utilization were similar at 4 months (eTable 2 in Supplement 2). Using multiple imputation did not substantially change the results (eTable 3 in Supplement 2).

**Table 2.** Risk of Health Care Utilization Outcome of Navigator Intervention Group vs Active Control Group

Outcome	Model 1 (N = 1300)^a^		Model 2 (N = 1300)^b^		Model 3 (n = 1116)^c^	
	HR (95% CI)	*P* value	HR (95% CI)	*P* value	HR (95% CI)	*P* value
ED visit within 1 y	0.96 (0.80-1.14)	.62	0.94 (0.78-1.12)	.48	0.98 (0.80-1.19)	.81
Hospitalized within1 y	0.59 (0.38-0.94)	.03	0.56 (0.35-0.90)	.02	0.59 (0.35-0.99)	.046

Abbreviations: ED, emergency department; HR, hazard ratio.

^a^ Model 1 was unadjusted.

^b^ Model 2 was adjusted for child age, child sex, child race/ethnicity, caregiver age, caregiver sex, baseline child health, baseline number of social risks, clinical site, clinic setting, and being asked about nonmedical needs in the past 12 months in a clinical setting.

^c^ Model 3 was additionally adjusted for poverty.

## Discussion

This study compared the utilization effects of 2 different clinical pediatrics interventions, both designed to better link families endorsing social risks with available social services. We found that during the 12 months after study enrollment, children enrolled in the trial’s patient navigator intervention had a 69% reduced risk of hospitalization than children in the active control group, but the intervention had no effect on ED visits. This translated to only 4.6% of patient navigator families having their child hospitalized vs 7.5% among the active control group.

To date, there have been few randomized clinical trials in pediatrics that have examined how an intervention designed to improve children’s social and environmental conditions might contribute to changes in acute health care utilization. Those that have looked at utilization outcomes typically blend the delivery of medical and social interventions, making it difficult to evaluate the added effect of addressing social needs. For example, in a study of a newborns living in low-income households in Boston, Sege et al^12^ randomly assigned families a family support specialist who provided both clinical education and social care assistance during routine clinic and home visits as well as via telephone, e-mail, or text. At 6 months after enrollment, infants in the intervention were less likely to have visited the ED than those in the control group. In contrast, our study presents results of an intervention that exclusively targeted families’ social needs and found no significant effects on ED visits. These differences in the effects on ED utilization may reflect the added value of the clinical intervention component in the study by Sege et al,^12^ although direct comparisons between study findings are complicated by the fact that the present study did not include a no-treatment control group.

We did find a differential effect of the navigator intervention vs active control on pediatric hospitalization. Conceptually, the mechanism for this effect is intuitive: decreased social risks may help families prioritize healthy behaviors, such as nutritious food or physical activity, or decrease unhealthy exposures, such as mold. However, work by Berkowitz et al^16^ suggests that the pathways through which reducing social risks improves health outcomes may be nonlinear. Our study does not clarify the pathways through which the intervention operated. Future research is needed to explore how addressing social needs contributes to changes in health and health care utilization.

Our study findings showing differential intervention effects on children’s acute care utilization patterns should inform health systems’ calculations on the return on investment of different social care programs. Although the navigation intervention may take more resources to initiate and sustain compared with the provision of written resource guides, it may nonetheless result in a higher return on investment, especially considering that the average cost of a nonbirth pediatric inpatient hospitalization is $13 400.^17^ However, future cost-effectiveness analyses are needed to inform this potential investment.

Since this study was conducted, the coronavirus disease 2019 pandemic has drastically changed how clinical and social care are delivered, at least temporarily. It is important to note that while the navigator intervention did offer the option of in-person meetings, it also consisted of telephone and email follow-up options. While providing the navigator intervention solely via telephone or email would need to be assessed before drawing conclusions about its efficacy, future work should explore this, given that it would allow navigators to connect with patients during periods that require shelter-in-place policies.

### Limitations

There are several study limitations. As described in the original publication,^9^ the low rate of study enrollment may have led to selection bias, whereby the types of families enrolling in the study were not a representative sample of the broader clinic populations from which the study was sampled. Additionally, families in the navigator intervention group endorsed a higher number of baseline social needs, although both the navigator intervention and active control groups reported a mean of more than 2 social needs at baseline.

We were also limited to data in the hospital systems with which the study sites were affiliated. While both sites serve as the safety-net hospitals in their respective cities, it is possible that families could have used health care at other clinical settings during the follow-up period or moved away from the study cities.

Finally, 28.1% of participating families either did not consent to access to child EHR data or had missing survey data related to these analyses; an additional 10.2% declined to provide income data, raising concerns that the sample analyzed here may differ from the overall sample. This could have led to overestimation or underestimation of intervention effects. For example, if families were hesitant to consent to having EHR data used because of lack of trust in the health care system, this might have also led them to distrust organizations referred to in the intervention, resulting in a lack of follow-up with community organizations and a potential reduction in study effects.

## Conclusions

In this randomized clinical trial based in pediatric acute and primary care settings, the services of a volunteer navigator, who worked with families longitudinally to help them connect with available social services, were associated with reduced risk of child hospitalization during the 12-month period following enrollment compared with those enrolled in an active control group. Social care programs in other pediatric settings could potentially result in large reductions in inpatient stays and associated cost savings. These findings could improve return on investment calculations for social care programs in pediatrics settings.

## 

## Notes

Supplement 1. **Trial Protocol**

Supplement 2. **eTable 1. **Characteristics of the Sample Excluded vs the Sample Analyzed **eTable 2.** Risk of Health Care Utilization Outcome of Navigator Intervention Group Compared With Active Control Group 4 Months After Enrollment **eTable 3.** Risk of Health Care Utilization Outcome of Navigator Intervention Group Compared With Active Control Group Using Multiple Imputation

Supplement 3. **Data Sharing Statement**
