# Supplementary material for: Effects of In-Person Navigation to Address Family Social Needs on Child Health Care Utilization: A Randomized Clinical Trial
Source: JAMA Netw Open. 2020 Jun 1;3(6):e206445. doi: 10.1001/jamanetworkopen.2020.6445 (PMC7265099; doi:10.1001/jamanetworkopen.2020.6445)
Supplement: Supplement 1. — Trial Protocol [file jamanetwopen-3-e206445-s001.pdf]

The study was registered with clinicaltrials.gov (NCT01939704).

- ☐ The section of your progress report if it provides the most current information about your human subjects work
- ☐ The grant is not attached. The study is funded by an award that does not describe specific plans for human subjects, such as career development awards (K awards), cooperative agreements, program projects, and training grants (T32 awards) OR UCSF (or the affiliate institution) is not the prime recipient of the award

8.0 Sites

8.1 Institutions (check all that apply):

- ☐ UCSF
- ☐ China Basin
- ☐ Helen Diller Family Comprehensive Cancer Center
- ☐ Mission Bay
- ☐ Mount Zion
- ☑ San Francisco General Hospital (SFGH)
- ☐ SF VA Medical Center (SF VAMC)
- ☐ Blood Centers of the Pacific (BCP)
- ☐ Blood Systems Research Institute (BSRI)
- ☐ Fresno (Community Medical Center)
- ☐ Gallo
- ☐ Gladstone
- ☐ Institute on Aging (IOA)
- ☐ Jewish Home
- ☐ SF Dept of Public Health (DPH)

8.2 Check all the other types of sites not affiliated with UCSF with which you are cooperating or collaborating on this project (Help Text updated 9/13):

- ☐ Other UC Campus
- ☐ Other institution
- ☐ Other community-based site
- ☐ Foreign Country

List the foreign country/ies:

8.3 Check any research programs this study is associated with:

- ☐ Cancer Center
- ☐ Center for AIDS Prevention Sciences (CAPS)
- ☐ Global Health Sciences
- ☐ Immune Tolerance Network (ITN)
- ☐ Neurosciences Clinical Research Unit (NCRU)
- ☐ Osher Center
- ☐ Positive Health Program

9.0 Study Design

9.1 * Study design (Help Text updated 9/13):

We are proposing a 4-arm, 18 month randomized control clinical trial to address social determinants of health, improve health status and health care satisfaction, and change health care utilization patterns in the Pediatric Urgent Care and Primary Care Clinics at SFGH. We will compare our CCLiP protocol to standard of care in both the Pediatric Primary Care Clinic & Urgent Care. All arms (intervention and control) include a baseline survey including social needs assessment, health care status, health care satisfaction and health care utilization assessment that is started in clinic and can be completed in clinic or by phone (social need priorities must be completed before leaving clinic); and two telephone-based follow up surveys at 6 weeks and 4 months. Patients in

the intervention arms (whether in urgent care or primary care) will receive a maximum of 3 months of intervention protocol which includes a 30 minute intervention at time of enrollment or subsequently by phone in addition to twice monthly follow-up phone calls for up to 3 months to help address social needs (see attached Algorithms). Patients in all arms will receive 6 weeks and 4-month follow-up surveys. A table of this study design is included below.

Pediatric Primary Care
Pediatric Urgent Care
Intervention
Baseline Survey + 30 minute intervention + biweekly follow up as necessary + 3 and 6 month follow-up
Baseline Survey + 30 minute intervention + biweekly follow up as necessary + 3 and 6 month follow-up
Control
Baseline Survey + 3 and 6 month follow-up
Baseline Survey + 3 and 6 month follow-up

9.2 If this is a clinical trial, check the applicable phase(s) (Help Text updated 9/13):

- ☐ Phase I
- ☐ Phase II
- ☐ Phase III
- ☐ Phase IV

10.0 Scientific Considerations

10.1 Hypothesis (Help Text updated 9/13):

This study has a hypothesis:

⦿Yes ❍No

If yes, state the hypothesis or hypotheses:

We hypothesize that by addressing the environmental and social factors that contribute to health within the setting of the medical home, we will be able to better connect families to community resources, enable more appropriate use of healthcare resources, improve health status and enhance patient satisfaction.

10.2 * List the specific aims:

CCLiP aims to evaluate the effects of family navigation. Specifically, we will measure the effects of the program on:

1. Family’s connection to community resources and public benefits
2. Caregiver satisfaction and connectedness to Children's Clinic at SFGH as a medical home
3. Child health care utilization
4. Child health care status.

10.3 Statistical analysis:

Power Analysis. Sample size and power estimates for comparison of participants from the control and intervention groups within each setting (Primary Care and Urgent Care) and comparison of intervention effects across the Primary Care and Urgent Care settings are based on alpha = 0.05 and 2-sided t-tests on change from baseline to 3 and 6 months. Estimates of means (SDs) at follow-up from related studies suggest effect sizes that are small to moderate, with 0.20 to .40 SD unit differences. The study is powered 80% to detect small effect sizes (Cohen’s d=.20).

Within each of the two settings (i.e., Primary Care and Urgent Care), a total sample of 1118 participants (n=559 assigned to Control group and 559 to intervention group) will provide power to exceed 0.80 for tests on the primary outcomes within each setting and between settings. We conservatively estimate a 30% attrition rate over the course of the study, resulting in a final sample of 394 participants in each group (Control or Intervention) per setting (Primary Care or Urgent Care). This equates to a total sample size of 788 from the Primary Care setting and a total sample of 788 from the Urgent Care setting. This sample size allows at least 80% power to detect estimated mean differences of changes from baseline of magnitude 0.20-0.40 for our Likert based continuous primary outcomes and power to detect a 10% percentage difference between the two intervention groups or between the intervention groups from the two settings on binary outcomes.

Descriptive Analyses & Missing Data. Descriptive analyses will include demographics, diabetes status, biological affective and management variables. Data will be inspected for outliers and out-of-range values. Examination of distributions may prompt transformations, where they are defensible and are a component of the best available analysis strategy. Although protocols have been designed to promote retention, some attrition is inevitable. We will perform analyses to determine whether differential attrition occurred by patient characteristics. Likelihood based approaches (e.g. GLMMs) will be used to handle missing data. This approach fits models to all available data and invoke the relative assumption that the data are missing at random.

Background demographics will be examined to describe the sample and will beincluded in multivariate analysis if they are related to the outcome at p<.2, differ between treatment arms, or associated with dropout. Correlations among variables within each area of study outcomes will be examined and redundant measures will be combined or eliminated to avoid multicollinearity.

Assessment of Outcomes: We will fit regression models for cross-sectional and longitudinal outcomes. Demographics will be compared for the two arms at baseline within and between the Primary Care and the Urgent Care setting. Comparisons between the four groups (Intervention and Control groups within the two settings of Primary Care and Urgent Care) on changes of the primary outcomes will be estimated with linear mixed models and generalized linear models with generalized estimating equations (e.g., SAS PROC MIXED ROC GENMOD, SAS Institute Inc., 1999). Generally, we seek to model longitudinally the trajectory of the primary outcomes as a function of time, intervention group assignment, and group-by-time interaction. We begin by fitting base models, those with minimum covariates and, for longitudinal models, the most restricted residual covariance structures. Additional covariates then will be considered. Similar models will be used to examine each of the primary outcomes outlined in the aims section of the application. Empirical contributions to model selection decisions will include reference to information criteria (e.g., Akaike's). We will examine change from baseline for each outcome as the dependent variable. In addition to testing the groups-by-time interaction term, custom contrasts will assess group differences within each of the two settings (Primary Care and Urgent Care) at each time point.

Descriptive Analyses & Missing Data. Descriptive analyses will include demographics, diabetes status, biological affective and management variables. Data will be inspected for outliers and out-of-range values. Examination of distributions may prompt transformations, where they are defensible and are a component of the best available analysis strategy. Although protocols have been designed to promote retention, some attrition is inevitable. We will perform analyses to determine whether differential attrition occurred by patient characteristics. Likelihood based approaches (e.g. GLMMs) will be used to handle missing data. This approach fits models to all available data and invoke the relative assumption that the data are missing at random.

Background demographics will be examined to describe the sample and will beincluded in multivariate analysis if they are related to the outcome at p<.2, differ between treatment arms, or associated with dropout. Correlations among variables within each area of study outcomes will be examined and redundant measures will be combined or eliminated to avoid multicollinearity.

10.4 If this study has undergone scientific or scholarly review, please indicate which entity performed the review:

☐ Cancer Center Protocol Review Committee (PRC) (Full approval is required prior to final CHR approval for cancer-related protocols.)

☐ CTSI Clinical Research Center (CRC) advisory committee

☐ Departmental scientific review

☐ Other:

Specify Other:

11.0 Background

11.1 Background:

Adverse social circumstances like hunger and food insecurity can have dramatic, negative impacts on the health of vulnerable children.(1-5) In safety-net settings, the prevalence of these adverse social circumstances are alarmingly high.(6-7) A 2007 study from Boston Medical Center pediatric emergency department found that over 97% of presenting families had at least one basic social need; over 48% of families reported that within the last 12 months they had either been threatened with or experience utility shut-offs; and over 30% of families said that they had either reduced the size of their meals or skipped meals because they did not have enough money for food.(8) In a study in pediatric urban primary care clinics, over 82% of families reported a least one social need.(9)

Despite the preponderance of social need in both emergency room and primary care safety net settings, the current standard of practice in pediatric medicine fails to address many of the health-related social problems of struggling families. Garg et al. (2007) showed significant gaps between primary care providers' beliefs that they

should include social screening topics in pediatric visits and routinized social screening behaviors.(10) Other researchers have demonstrated that comprehensive social screening (across multiple social domains) is particularly rare in pediatric settings.(9)The evidence suggesting irregular and informal screening for health-related social needs belies studies demonstrating that conducting social screening informally during clinical encounters poorly estimates prevalence; providers routinely underestimate social needs in all areas except drug and alcohol use and need for nursing home placement.(11) Furthermore, standardized screening has been shown to be more sensitive than provider clinical interviews.(12)

There is growing evidence that patients who receive public benefits may have better health status and decreased emergency room utilization.(13-19). Despite this emerging literature that serves as important background for FIND implementation, existing intervention programs to address social factors in pediatric clinical settings (e.g. healthleadsusa.org) have not yet included scientific evaluations in either emergency room or primary care settings of program impacts on pediatric health care status or health care utilization, nor on patient satisfaction/connectedness to health care organizations. As a result of conducting a randomized controlled clinical trial, we hope to prove that by systematically addressing our families social determinants of health, we will better connect families to community resources, use healthcare resources more appropriately, improve health status and enhance patient satisfaction.

The CCLiP intervention specifically includes implementing a navigation desk at the SFGH Primary Care Clinic and Urgent Care. All families entering either site will be screened for social issues affecting their health, including nutrition, housing and financial insecurity. Families with a positive screen will be referred to CCLiP. CCLiP navigators will assist in addressing each family's needs. Using a client management database, CCLiP navigators will follow the family over time to ensure that the identified need has been successfully addressed. Using the RCT methodololgy described below, the users and non users of CCLiP will be compared over six months to examine outcomes including social needs, health status, connectedness to a medical home, and health care utilization.

References
See below.

11.2 Preliminary studies:

Patient surveys at SFGH have shown that almost 50% of families accessing care in the primary care clinics have experienced food insecurity over the last 12 months. This population is expected to be assisted with the CCLiP intervention. A pilot program has shown that this model can be successfully incorporated into clinic flow.

11.3 References:

1. Larson K, Russ SA, Crall JJ, Halfon N. Influence of multiple social risks on children's health. Pediatrics. 2008; 121;337.
2. Wood PR, Smith LA, Romero D, Bradshaw P, Wise PH, Chavkin W. Relationships between welfare status, health insurance status and health and medical care among children with asthma. Am J Pub Health. Sep 2002;92(9):1146-1452.
3. Cook JT, Frank DA, Berkowitz C, Black MM, Casey PH, Cutts DB, Meyers AF, Zaldivar N, Shalicky A, Levenson S, Heeren T. Welfare reform and the health of young children: A sentinel survey in 6 US cities. Arch PediatrAdolesc Med Jul 2002;156:678-684.
4. Frank DA, Neault NB, Skalicky A, Cook, JT, Wilson JD, Levenson S, Meyers AF, Heeren T, Cutts DB, Casey PH, Black MM, Berkowitz C. Heat or eat: the Low Income Home Energy Assistance Program and nutritional health risks among childen less than 3 years of age. Pediatrics 2006 Nov;118(5):e1293-302.
5. Meyers A, Cutts D, Frank DA, et al. Subsidized housing and children's nutritional status: data from a multisite surveillance study. Arch Pediatr Adolesc Med. 2005 Jun;159(6):551-6.
6. Garg A, Butz AM, Dworkin PH, Lewis RA, Serwint JR. Screening for basic social needs at a medical home for low-income children. ClinPediatr.2009 48:32-36;
7. Lawton E, Leiter K, Todd J, Smith L. Welfare reform: advocacy and intervention in the health care setting. Pub Health Reports. Nov/Dec 1999;114:540-549.
8. Hanson M, Lawton E. Between a rock and a hard place: The prevalence and severity of unmet legal needs in the pediatric emergency department setting. Medical Legal Partnership for Children. 2007.
9. Fleegler EW, Lieu TA, Wise PH, Muret-Wagstaff S. Families' health-related social problems and missed referral opportunities. Pediatrics. 2007;119:e1332–41.
10. Garg A, Butz AM, Dworkin PH, Lewis RA, Thompson RE, Serwint JR, Improving the management of family psychosocial problems at low-income children's well-child care visits: the WE CARE project. Pediatrics. 2007;120:547-558.
11. Bikson, K., McGuire, J.,Blue-Howells, J.&Seldin-Sommer, L. Psychosocial Problems in primary care: Patient and provider perceptions. Social Work in Health Care. 2009;48:736-749.
12. Keller, D., Jones, N., Savageau, J. A. &Cashman, S. B. Development of a brief questionnaire to identify families in need of legal advocacy to improve child health. Ambulatory Pediatrics. 2008;8:266-269.
13. Cook JT, Frank DA, Berkowitz C, Black MM, Casey PH, Cutts DB, Meyers AF, Zaldivar N, Skalicky A, Levenson S, Heeren T. Welfare reform and the health of young children: a sentinel survey in 6 US cities. Arch Pediatr Adolesc Med2002 Jul;156(7):678-84.
14. Frank DA, Neault NB, Skalicky A, Cook JT, Wilson JD, Levenson S, Meyers AF, Heeren T, Cutts DB, Casey PH, Black MM, Berkowitz C.Heat or eat: the Low Income Home Energy Assistance Program and nutritional and

health risks among children less than 3 years of age. Pediatrics 2006 Nov;118(5):e1293-302.
15. Meyers A, Cutts D, Frank DA, Levenson S, Skalicky A, Heeren T, Cook J, Berkowitz C, Black M, Casey P, Zaldivar N. Subsidized housing and children's nutritional status: data from a multisite surveillance study. Arch Pediatr Adolesc Med 2005 Jun;159(6):551-6.
16. Black MM, Cutts DB, Frank DA, Geppert J, Skalicky A, Levenson S, Casey PH, Berkowitz C, Zaldivar N, Cook JT, Meyers AF, Herren T. Special Supplemental Nutrition Program for Women, Infants, and Children participation and infants' growth and health: a multisite surveillance study. Pediatrics 2004 Jul;114(1):169-76.
17. Jones SJ, Jahns L, Laraia BA, Haughton B. Lower risk of overweight in school-aged food insecure girls who participate in food assistance: results from the panel study of income dynamics child development supplement. Arch Pediatr Adolesc Med 2003 Aug;157(8):780-4.
18. Cook JT, Frank DA, Berkowitz C, Black MM, Casey PH, Cutts DB, Meyers AF, Zaldivar N, Skalicky A, Levenson S, Heeren T, Nord M. Food insecurity is associated with adverse health outcomes among human infants and toddlers. J Nutr 2004 Jun;134(6):1432-8.
19. Cook JT, Frank DA, Levenson SM, Neault NB, Heeren TC, Black MM, Berkowitz C, Casey PH, Meyers AF, Cutts DB, Chilton M. Child food insecurity increases risks posed by household food insecurity to young children's health. J Nutr 2006 Apr;136(4):1073-6.

If you have a separate bibliography, attach it to the submission with your other study documents.

12.0 Sample Size and Eligibility

12.1 Number of subjects that will be enrolled at UCSF and affiliated institutions:

2250

12.2 Total number of subjects that will be enrolled at all sites (Help Text updated 9/13):

2250

12.3 Estimated number of people that you will need to consent and screen here (but not necessarily enroll) to get the needed subjects:

2250

12.4 Explain how and why the number of subjects was chosen (Help Text updated 9/13):

Power calculations, as indicated in the scientific considerations section of this application, have been based on an effort to detect a small effect size (Cohen's d=.20) between the intervention and control arms of the study. Power calculations already include estimated attrition.

12.5 * Eligible age range(s):

☑ 0-6 years
☑ 7-12 years
☑ 13-17 years
☑ 18+ years

12.6 Inclusion criteria:

Inclusion Criteria:
- English or Spanish speaking
- Parent/caregiver accompanying an SFGH Primary Care or Urgent Care Clinic patient 0-17 years old
- Consenting adult over or equal to 18 years old

12.7 Exclusion criteria:

Exclusion Criteria:
- Non-English or non-Spanish speaking caregiver
- Caregiver under age 18

- Caregiver accompanying patient is not familiar with the child’s living situation
- Has participated in study previously
- Has worked with the FIND in primary care setting
- Non Alameda County

Exclusion Criteria
- Non-English or non-Spanish speaking caregiver
- Caregiver under age 18
- Caregiver accompanying patient is not familiar with the child’s living situation
- Family participated in study previously
- Non-San Francisco resident
- Foster child or child in clinic for a child protective clearance exam

- Non Alameda County resident resident

Exclusion Criteria:
- Non-English or non-Spanish speaking caregiver
- Caregiver under age 18
- Caregiver accompanying patient is not familiar with the child’s living situation
- Has participated in study previously
- Has worked with the FIND in primary care setting
- Non Alameda County resident

Exclusion Criteria:
- Non-English or non-Spanish speaking caregiver
- Caregiver under age 18
- Caregiver accompanying patient is not familiar with the child’s living situation
- Has participated in study previously
- Has worked with the FIND in primary care setting
- Non Alameda County resident

12.8 There are inclusion or exclusion criteria based on gender, race or ethnicity:

❍Yes ⦿No

If yes, please explain the nature and rationale for the restrictions:

13.0 Other Approvals and Registrations

13.1 * Do any study activities take place on patient care units:

⦿Yes ❍No

If Yes, attach a letter of support for the study from the involved patient care manager(s).

13.2 * Does your protocol involve any radiation exposure to patients/subjects? The UCSF Radiation Safety Committee requires review of your protocol if it includes administration of radiation as part of standard of care OR research exposures:

❍Yes ⦿No

13.3 * This study may generate genetic data that may be broadly shared (e.g. submitted to NIH for Genome-Wide Association Studies (GWAS) in dbGaP, TCGA, etc):

❍Yes ⦿No

13.4 * This study involves administration of vaccines produced using recombinant DNA technologies to human subjects:

❍Yes ⦿No

13.5 * This study involves human gene transfer (NOTE: Requires NIH Recombinant DNA Advisory Committee (RAC) review prior to CHR approval):

❍Yes ⦿No

13.6 This study involves other regulated materials and requires approval and/or authorization from the following regulatory committees:

☐ Institutional Biological Safety Committee (IBC)

Specify BUA #:

☐ Institutional Animal Care and Use Committee (IACUC)

Specify IACUC #:

☐ Radiation Safety Committee

Specify RUA #:

☐ Radioactive Drug Research Committee (RDRC)

Specify RDRC #:

☐ Controlled Substances

14.0 Procedures

14.1 * Procedures/Methods (Help Text updated 9/13) For clinical research list all study procedures, test and treatments required for this study, including when and how often they will be performed. If there are no clinical procedures, describe the Methods:

Research assistants will ask caregivers visiting in Primary Care or Urgent Care if they are interested in participating in the Community to Clinic Linkage study using a prewritten script. Any patient caregiver visiting Primary Care Clinic or Urgent Care can be approached for eligibility screening. Randomization in this study is by day of the week, so randomization assignment by group is known by Research Staff at the beginning of each shift. (See Introduction Script attached.)

Willing participants will be assigned an eligibility screening ID # and screened for eligibility by research assistant in the clinic room using an Eligibility Survey. .

Eligible participants will be assigned an Enrollment ID in a master file kept outside of Qualtrics in a password protected Excel file on the an encrypted and password protected laptop (see Study Eligibilty and Enrollment templates.)

Research Assistant will review consent (labeled with Study ID) with eligible caregivers (as described above). Consents to participate will include willingness to provide complete caregiver name, phone number, address and email address. (See CCLIP Evaluation Flow Diagram attached.) Consenting caregivers will be given a copy of the consent form. They will also be asked to review and sign a HIPAA authorization form.

Consenting participants will have study ID number linked to child medical record number. (NB: Any duplicate MRN will not be eligible for participation.)

For families enrolled prior to use of HIPAA Authorization forms, we will send a letter outlining reason for adding HIPAA authorization, data to be collected from medical record, and HIPAA Authorization form via mail along with a pre-stamped, pre-addressed envelope asking them to sign and return. This letter will be sent to all enrolled families in both arms of the study.

At 6 and 12 months, we will extract visit data from SFGH LCR on all enrolled patients to look at differences in health care utilization between enrolled families in the two arms of the study.

· Control group:

a. Control group will complete baseline survey. If positive social needs on baseline survey (survey electronically flags Research Assistants with information re: what positive items were and priorities for those items), patients will be given a list of county-of-residence-specific social service resources (2-1-1 card).

b. Control group will be contacted by phone (see Telephone Follow Up Script attached) by the research assistant at 6 weeks and again at 4 months for follow-up surveys, each expected to last approximately 15 minutes. After completion of each follow up questionnaire, the subject will be paid $5 in Target gift cards. Cards will be mailed or emailed to the subject based on their preference.

· CCLiP Intervention group

a. Intervention group will complete baseline survey. If respondents do not meet criteria for intervention (criteria include positive social needs assessment on at least 1 item in attached Baseline Survey), respondents will be contacted by phone by the research assistant at 6 weeks and again at 4 months for follow-up surveys, each expected to be approximately 15 minutes.

b. If caregiver meets criteria for intervention (positive social needs on at least 1 item in Baseline Survey (survey electronically flags Research Assistant with information re: what positive items were and ranked priority list for those items) respondents in intervention group will meet with Research Assistant in Primary Care Clinic/ Urgent Care or by phone for approximately 30 minutes to review Social Services Intervention Algorithms. (See CCLIP Algorithms attached.)

c. Following Intervention, caregiver and Research Assistant complete Social Services Intervention Contract (see CCLIP Intervention Contract attached) listing follow-up recommendations (both for caregiver and for CCLiP Staff/Research Assistant) when initial intervention completed.

d. Research Assistant completes Qualtrics-based survey re: interventions or referrals made (internal, external referrals, information sheets, etc.—see attached Staff Survey) related to each social needs priority area. The purpose of this brief information capture survey from Research Assistant is to ensure some information about initial intervention is captured in case participant is lost to follow-up. The Research Assistant also enters patient tracking data into password-protected Excel spreadsheet (see CCLiP Study Day Log), including caregiver name, patient first name only, phone number, address, and priority social needs, as well as interventions administered and recommended follow-up items (in Field Notes #1 section.)
e. Research Assistant follows-up with caregivers for 5-30 minute phone visits every 2 weeks until prioritized social needs met, three months after initial visit, end of algorithm reached, or caregiver requests discontinuation. Intervention information and outcomes are entered into "Field Notes" Excel file described above.
f. Research Assistant follows-up with caregivers at 6 weeks and 4 months to complete telephone-administered survey. (See attached Telephone Follow-up Script and 6 weeks and 4 mo Follow-up Survey.) After each follow up survey the participant will receive a $5 Target gift card. Cards will be mailed or emailed to the subject based on their preference.
Eligibility will be determined via a Qualtrics eligibility screening survey administered by the research assistant (RA). The survey covers the inclusion and exclusion criteria as listed above.

If you have a procedure table, attach it to the submission with your other study documents.

14.2 Interviews, questionnaires, and/or surveys will be administered or focus groups will be conducted:

☉Yes ❍No

List any standard instruments used for this study:

Patients eligible for enrollment, who consent to be in the study, will be subject to:

a. Baseline social, health status and health care utilization survey conducted via laptop using Qualtrics. (See attached Baseline Survey.) Survey can be administered through via laptop or read to low-literacy patients in English or in Spanish. Research assistants will be available at all times.
b. Intervention administered by Research Assistant first in clinical setting, though ok to complete by phone if necessary, then in twice monthly telephone follow-up (every two weeks) per algorithms attached. Research Assistant will complete Qualtrics survey after initial interview to describe any interventions provided at first visit.
c. Phone follow-up surveys will be conducted at 6 weeks and 4 months. Research Assistant will call or email a maximum of 3 times to arrange or conduct phone follow-up. If there is no response to the third phone calls, patients will receive a letter addressed to the address of record asking them to phone the research line if they are willing to complete the study survey. Each survey is anticipated to last a maximum of 15 minutes. After each follow up survey the participant will be mailed a $5 Target gift card, for a total of $10 of Target gift cards. Cards will be mailed or emailed to the subject based on their preference.

All Research Assistants will complete CITI and HIPAA training in addiiton to receiving training in research/consent rules and regulations, cultural sensitivity, legal and and social needs screening and intervention, as well as survey administration techniques.

Attachments
- Evaluation flow diagram #1
- Eligibility survey #2
- Baseline survey #3
- Algorithms by social need #4
- Social Needs Intervention Contract #5
- Staff Intervention survey #6

Follow-up surveys (6 weeks and 4 months) #7

Attach any non-standard instruments at the end of the application.

14.3 Conduct of study procedures or tests off-site by non-UCSF personnel:

❍Yes ☉No

If yes, explain:

14.4 Sharing of experimental research test results with subjects or their care providers:

❍Yes ☉No

If yes, explain:

14.5 * Specimen collection for future research and/or specimen repository/bank administration:

❍Yes ⦿No

14.6 Time commitment (per visit and in total):

Control Group
Approximately 15 minute baseline survey (in-person/computer assisted)
Approximately 15 minute survey at 6 weeks (phone)
Aproximlately 15 minute survey at 4 months (phone)
Intervention Group
Approximately 15 minute baseline survey (in-person/computer assisted)
Approximately 30 minute intervention (in-person or by phone)
Approximately 5-30 minute bi-weekly phone calls as needed up to 3 months
Approximately 15 minute survey at 6 weeks (phone)
Aproximlately 15 minute survey at 4 months (phone)

14.7 Locations:

SFGH Pediatric Urgent Care and Primary Care clinics located on 6M main hospital.

14.8 Describe the resources in place to conduct this study in a way that assures protection of the rights and welfare of participants:

1. All study participants are required to review and sign informed consent prior to participation.
2. All data are stored on secure servers.
3. All staff required to complete HIPAA and CITI training, in addition to legal, cultural sensitivity training.
4. A limited number of study staff will have access to study data.
5. PHI will be linked by study ID to main data files and only accessed as needed by research assistants with clearance to view pertinent files.
6. Research assistants will not have access to child medical records.

15.0 Risks and Benefits

15.1 * Risks and discomforts:

We do not intend to disclose any confidential information that caregivers provide. Caregivers will be informed, however, that health care personnel are required by law to report child abuse. If an answer suggests that a patient is in danger, the research staff will notify the attending physician in the Urgent Care Clinic/Primary Care Clinic.

15.2 Steps taken to minimize risks to subjects:

There are no risks for participating in this survey aside from the bounds of confidentiality. Also see Section 16.8.

15.3 Benefits to subjects:

⦿Yes ❍No

If yes, describe:

We believe that attending to families' social and environmental needs will improve health status and utilization for patients. Connecting patients to available community resources is a perceived benefit.

15.4 Benefits to society:

If our hypothesis is correct, then connecting patients to available community resources should decrease inappropriate health care utilization, increase caregivers' ability to participate in community activities, including employment opportunities, and contribute a net benefit to society.

15.5 Explain why the risks to subjects are reasonable:

We feel the benefits of this study far outweigh the minimal risks. With the information from the study, we will be able to better provide care to our patients and better understand how to effectively address social determinants of health.

16.0 Confidentiality and Privacy

16.1 Plans for maintaining privacy in the research setting:

The consent form will stipulate that information provided by the subjects will remain strictly confidential, with access limited to the research staff. Names will be separated from all recorded materials and code numbers will be inserted. No one but the project staff will have access to the master list linking subjects' full names to code numbers, and all information obtained will be coded. The master list will be stored in an Excel file that is password protected in firewalled computer files with access limited only to project investigators and appropriate research staff. Signed consents will also be in a locked filing cabinet in a locked office in the Primary Care Clinic/ Urgent Care. Access to the Qualtrics system will be through the use of log-ins and passwords, with patient names or other PHI excluded. Research staff will only have access to de-identified datasets. Publications or presentation of findings will not include information identifying the subjects.
Data collected from Qualtrics will be identified through a patient number only and is accessed through a secure website.

Only research staff will have access to the master list linking subjects' full names to code numbers. In cases where there is immediate danger to self or others (including patient) data will be conveyed to attending physician in the Primary Care Clinic & Urgent Care as required by law. Following protocols, this information about immediate danger may also be submitted to the on-call social worker pending Primary Care Clinic & Urgent Care Attending approval.

The master file linking study data with PHI that will be kept on the UCSF server will be destroyed at the end of the study.

The following people will have access to the master linking file:
Ellen Laves
Amy Whittle
Abby Burns
Anais Amaya

16.2 Possible consequences to subjects resulting from a loss of privacy:

Caregivers' household information on social and environmental needs is included in the study dataset. A privacy breech that includes this information is unlikely to have any negative repercussions other than potential social stigma.

16.3 Study data are:

- ☐ Derived from the Integrated Data Repository (IDR) or The Health Record Data Service (THREDS) at SFGH
- ☑ Derived from a medical record (e.g. APeX, OnCore, etc. Identify source below)
- ☐ Added to the hospital or clinical medical record
- ☐ Created or collected as part of health care
- ☐ Used to make health care decisions
- ☑ Obtained from the subject, including interviews, questionnaires
- ☐ Obtained from a foreign country or countries only
- ☐ Obtained from records open to the public
- ☐ Obtained from existing research records
- ☐ None of the above

If derived from a medical record, identify source:

SFGH LCR

16.4 Identifiers may be included in research records:

⦿Yes ❍No

If yes, check all the identifiers that may be included:

- ☑ Names
- ☑ Dates
- ☐ Postal addresses
- ☑ Phone numbers

- [ ] Fax numbers
- [x] Email addresses
- [ ] Social Security Numbers*
- [x] Medical record numbers
- [ ] Health plan numbers
- [ ] Account numbers
- [ ] License or certificate numbers
- [ ] Vehicle ID numbers
- [ ] Device identifiers or serial numbers
- [ ] Web URLs
- [ ] IP address numbers
- [ ] Biometric identifiers
- [ ] Facial photos or other identifiable images
- [x] Any other unique identifier

* Required for studies conducted at the VAMC

16.5 Identifiable information might be disclosed as part of study activities:

○ Yes ⦿ No

If yes, indicate to whom identifiable information may be disclosed:

- [ ] The subject's medical record
- [ ] The study sponsor
- [ ] Collaborators
- [ ] The US Food & Drug Administration (FDA)
- [ ] Others (specify below)
- [ ] A Foreign Country or Countries (specify below)

If Others, specify:

16.6 Indicate how data are kept secure and protected from improper use and disclosure (check all that apply): NOTE: Whenever possible, do not store subject identifiers on laptops, PDAs, or other portable devices. If you collect subject identifiers on portable devices, you MUST encrypt the devices.

- [x] Data are stored securely in My Research
- [x] Data are coded; data key is destroyed at end of study
- [x] Data are coded; data key is kept separately and securely
- [ ] Data are kept in a locked file cabinet
- [ ] Data are kept in a locked office or suite
- [x] Electronic data are protected with a password
- [x] Data are stored on a secure network
- [ ] Data are collected/stored using REDCap or REDCap Survey
- [ ] Data are securely stored in OnCore

16.7 Additional measures to assure confidentiality and protect identifiers from improper use and disclosure, if any:

PHI on pediatric patients and master file linking study ID to medical record numbers will be kept separately from research survey data and protected on secured encrypted, password-protected laptop with password-protected files. PHI information is only used for intervention and control follow-up (i.e. to reach participants after initial visit.) Data analysis will be based on data from research surveys that only include study ID numbers.

16.8 This study may collect information that State or Federal law requires to be reported to other officials or ethically requires action:

⦿ Yes ○ No

Explain:

If respondents disclose information indicating child abuse or neglect, research assistants are required by law to report this to the attending physician.

16.9 This study will be issued a Certificate of Confidentiality:

❍Yes ⦿No

17.0 Subjects

17.1 Check all types of subjects that may be enrolled:

☐ Inpatients
☑ Outpatients
☐ Healthy volunteers
☐ Staff of UCSF or affiliated institutions

17.2 Additional vulnerable populations:

☑ Children
☐ Subjects unable to consent for themselves
☐ Subjects unable to consent for themselves (emergency setting)
☐ Subjects with diminished capacity to consent
☑ Subjects unable to read, speak or understand English
☐ Pregnant women
☐ Fetuses
☐ Neonates
☐ Prisoners
☑ Economically or educationally disadvantaged persons
☐ Investigators' staff
☐ Students

Explain why it is appropriate to include the types of subjects checked above in this particular study:

We are conducting this study with caregivers of patients in the SFGH Peds Urgent Care and Primary Care Clinics, where many caregivers are unable to read/write English, and many are socioeconomically disadvantaged. Since the aim of the study is to better connect patients who need resources with available community services, it is essential to recruit these families. The study is aimed at improving pediatric health outcomes so recruiting from pediatric populations is an additional requirement. We have checked the "children" box because we are accessing medical records of the index child (child receiving care at SFGH on day of enrollment) at 12 months following enrollment.

Describe the additional safeguards that have been included in the study to protect the rights and welfare of these subjects and minimize coercion or undue influence:

We are consenting caregivers who have knowledge of the pediatric patients' living environment. This should minimize risks to the rights and welfare of pediatric patients. We are adding an adolescent assent requirement for using EMR utilization data at 12 months following household enrollment in the study.

18.0 Inclusion of Children in Research

18.1 This study will enroll children who can legally consent for themselves:

❍Yes ⦿No

If yes, explain why they can consent for themselves in the research setting:

If you will ONLY be enrolling children who can legally consent for themselves, press SAVE and CONTINUE to skip the rest of this section.

18.2 Select all the regulatory categories that apply:

☑ No greater than minimal risk (45 CFR 46.404, 21 CFR 50.51)

☐ Greater than minimal risk but presenting prospect of direct benefit (45 CFR 46.405, 21 CFR 50.52)

☐ Greater than minimal risk (though only a minor increase over minimal risk) and no prospect of direct benefit but likely to yield generalizable knowledge about the subjects disorder or condition (45 CFR 46.406, 21 CFR 50.53)

☐ Research not otherwise approvable which presents an opportunity to understand, prevent, or alleviate a serious problem affecting the health or welfare of children (45 CFR 46.407, 21 CFR 50.54)

Explain why the research in this study falls under the above category or categories:

18.3 Parental permission or waiver:

☑ Parental permission will be obtained

☐ Waiver of parental permission is requested: Parental permission is not a reasonable requirement

☐ Waiver of parental permission is requested: The waiver meets the provisions for a waiver of consent set forth in 45 CFR 46.116, Subpart A

If you are requesting a waiver of parental permission, explain why the study meets the regulatory criteria for this waiver:

18.4 Assent of children or waiver:

☑ Assent of children old enough to provide assent will be obtained

☐ Waiver of assent is requested: Children cannot be consulted or the research has prospect of direct benefit only available in the study

☐ Waiver of assent is requested: The waiver meets the provisions for a waiver of consent set forth in 45 CFR 46.116, Subpart A

If you are requesting a waiver of child's assent, explain why the study meets the regulatory criteria for this waiver:

18.5 Documentation of permission and assent (select all that will be used):

☑ Permission form addressed to the parents

☐ Simplified assent form addressed to the child, 7-12 years old (parents get separate form)

☑ Assent form addressed to the child, 13 years and older (for subjects and parents)

☐ Assent form addressed to the child, 13 years and older (parents get separate form)

Check one:

⦿ One parent's signature will be obtained

❍ Two parents' signatures will be obtained

If this study is approvable under .404 or .405 and you plan to get permission from only one parent, explain why you think one parent's permission is sufficient:

The study poses minimal risk to children and caregivers.

18.6 This study may enroll wards of the state:

❍ Yes ⦿ No

19.0 Inclusion of Non-English Speaking Subjects

19.1 Indicate which method(s) you will use to consent non-English speaking subjects:

☑ Preferred Method—Consent form and other study documents will be available in the subject’s primary language Personnel able to discuss participation in the patient’s language will be present for the consent process.

☐ Short-Form—A qualified interpreter will translate the consent form verbally, and subjects will be given the Experimental Subject's Bill of Rights in their primary language, following instructions in Those Who do not Read, Speak or Understand English for required witnessing and signatures

19.2 Explain how you will maintain the ability to communicate with non-English speakers throughout their participation in the study:

We plan to have a fully bilingual, bicultural trained research assistant available during study hours to communicate with Spanish-speaking participants. The consent document will be available in English and in Spanish. Interpreter services will not be used to consent patients for this study.

20.0 Recruitment

20.1 * Methods (check all that apply):

☑ Study investigators (and/or affiliated nurses or staff) recruit their own patients directly in person or by phone.

☐ Study investigators recruit their own patients by letter. Attach the letter for review.

☐ Study investigators send a "Dear Doctor" letter to colleagues asking for referrals of eligible patients. If interested, the patient will contact the PI or the PI may directly recruit the patients (with documented permission from the patient). Investigators may give the referring physicians a study information sheet for the patients.

☐ Study investigators provide their colleagues with a "Dear Patient" letter describing the study. This letter can be signed by the treating physicians and would inform the patients how to contact the study investigators. The study investigators may not have access to patient names and addresses for mailing

☐ Advertisements, notices, and/or media used to recruit subjects. Interested subjects initiate contact with study investigators. Attach ads, notices, or media text for review. In section below, please explain where ads will be posted.

☐ Study investigators identify prospective subjects through chart review. (Study investigators request a Waiver of Authorization for recruitment purposes.)

☐ Large-scale epidemiological studies and/or population-based studies: Prospective subjects are identified through a registry or medical records and contacted by someone other than their personal physician. (Study investigators request a Waiver of Authorization for recruitment purposes.)

☐ Direct contact of potential subjects who have previously given consent to be contacted for participation in research. Clinic or program develops a CHR-approved recruitment protocol that asks patients if they agree to be contacted for research (a recruitment database) or consent for future contact was documented using the consent form for another CHR-approved study.

☐ Study investigators list the study on the School of Medicine list of UCSF Clinical Trials website or a similarly managed site. Interested subjects initiate contact with investigators.

☐ Study investigators recruit potential subjects who are unknown to them through methods such as snowball sampling, direct approach, use of social networks, and random digit dialing.

☐ Other

If Other, explain:

20.2 * How, when, and by whom eligibility will be determined:

Patients and their caregivers will be asked at they time they are roomed by the nurse or medical assistant if they are willing to talk with a research assistant about an ongoing research study on social needs that families may have outside of their immediate health concerns and ways to address those needs.

20.3 * How, when, where and by whom potential subjects will be approached:

If caregiver assents to talk with research assistant, they will be approached by the research assistant in the clinic room to assess eligibility and review informed consent.

20.4 * Protected health information (PHI) will be accessed prior to obtaining consent:

⦿Yes ❍No

21.0 Waiver of Consent/Authorization for Recruitment Purposes This section is required when study investigators (and/or affiliated nurses or staff) recruit their own patients directly.

21.1 * Study personnel need to access protected health information (PHI) during the recruitment process and it is not practicable to obtain informed consent until potential subjects have been identified:

☉Yes

If no, a waiver of consent/authorization is NOT needed.

21.2 * A waiver for screening of health records to identify potential subjects poses no more than minimal risk to privacy for participants:

☉Yes

If no, a waiver of authorization can NOT be granted.

21.3 * Screening health records prior to obtaining consent will not adversely affect subjects' rights and welfare:

☉Yes

If no, a waiver of authorization can NOT be granted.

21.4 * Check all the identifiers that will be collected prior to obtaining informed consent:

☑ Names
☐ Dates
☐ Postal addresses
☐ Phone numbers
☐ Fax numbers
☐ Email addresses
☐ Social Security Numbers*
☐ Medical record numbers
☐ Health plan numbers
☐ Account numbers
☐ License or certificate numbers
☐ Vehicle ID numbers
☐ Device identifiers or serial numbers
☐ Web URLs
☐ IP address numbers
☐ Biometric identifiers
☐ Facial photos or other identifiable images
☐ Any other unique identifier
☐ None

Note: HIPAA rules require that you collect the minimum necessary.

21.5 * Describe any health information that will be collected prior to obtaining informed consent:

No records will actually be collected by study personnel before informed consents are obtained. Study staff will ask re: caregiver willingness to participate in study activities.

Note: HIPAA requires that you collect the minimum necessary.

21.6 * Describe your plan to destroy the identifiers at the earliest opportunity consistent with the research or provide a health or research justification for retaining the identifiers, or indicate and explain that retention is required by law:

Identifiers will be destroyed at study end by erasing all electronic linking files.

22.0 Informed Consent

22.1 * Methods (check all that apply):

☑ Signed consent will be obtained from subjects and/or parents (if subjects are minors)
☐ Verbal consent will be obtained from subjects using an information sheet or script
☐ Electronic consent will be obtained from subjects via the web or email
☐ Implied consent will be obtained via mail, the web or email
☐ Signed consent will be obtained from surrogates
☐ Emergency waiver of consent is being requested for subjects unable to provide consent
☐ Informed consent will not be obtained

22.2 * Process for obtaining informed consent:

a) The consent will take place in exam rooms in SFGH Pediatric Primary Care Clinic & Urgent Care Clinic.
b) Families will be able to ask questions of the fully bilingual/bicultural Eng/Spanish research assistants before signing consents. They will be able to participate in the study or decline and their decision will not affect the quality of care that they receive.
c) We will have written material in both languages.
d) For families enrolled prior to addition of HIPAA Authorization Forms, we will send letter explaining request to add HIPAA information to outcomes. This letter will accompany a revised consent and a HIPAA Authorization form along with a stamped, pre-addressed envelope. When sent to families where enrolled adolescent was 13-17 years old, we will also include an adolescent assent form.

22.3 * How investigators will make sure subjects understand the information provided to them:

We will have bilingual research assistants available to answer any questions.

23.0 Financial Considerations

23.1 Subjects payment or compensation method (check all that apply):

Payments will be (check all that apply):

☐ Subjects will not be paid
☐ Cash
☐ Check
☐ Debit card
☑ Gift card
☐ Reimbursement for parking and other expenses
☐ Other:

Specify Other:

23.2 Describe the schedule and amounts of payments, including the total subjects can receive for completing the study. If deviating from recommendations in Subject Payment Guidelines, include specific justification below.

Each participant will be paid up to $10 for taking part in this study. After each phone follow-up survey (at approximately 6 weeks and 4 months after enrollment), we will send the participant a Target gift card of $5. It will be sent by mail or by email, whichever the participant prefers.

23.3 Costs to Subjects: Will subjects or their insurance be charged for any study procedures?

○ Yes ⦿ No

If yes, describe those costs below, and compare subjects' costs to the costs associated with alternative care off-study. Finally, explain why it is appropriate to charge those costs to the subjects.

24.0 CTSI Screening Questions

24.1 * This study will be carried out at one of the UCSF Clinical Research Services (CRS) centers or will utilize CRS services. CRS centers are at the following sites: SFGH Clinical Research Center Moffitt Adult Clinical Research

Center Moffitt Hospital Pediatrics & NCRC Mount Zion Hospital Clinical Research Center Tenderloin Center CHORI Children's Hospital Pediatrics & Adult Clinical Research Center Kaiser Oakland Research Unit SF VA Medical Center Clinical Research Unit   Please note: Effective 3/1/14, the CRS form will no longer be completed and submitted in iRIS. The CRS budget request form can be found at: https://accelerate.ucsf.edu/files/crs/BudgetRequest2015.docx. Follow the instructions on the form to submit.   Even if you click 'Yes' to this question, the form will no longer proceed to the Clinical Research Services (CRS) Application Form section.

❍Yes ⊙No

24.2 This project involves community-based research:

❍Yes ⊙No

24.3 This project involves practice-based research:

❍Yes ⊙No

25.0 End of Study Application

25.1 End of Study Application Form   To continue working on the Study Application: Click on the section you need to edit in the left-hand menu. Remember to save through the entire Study Application after making changes.   If you are done working on the Study Application: Click Save and Continue. If this is a new study, you will automatically enter the Initial Review Submission Packet form, where you can attach consent forms or other study documents. Review the Initial Review Submission Checklist for a list of required attachments.   Answer all questions and attach all required documents to speed up your approval.
