# Supplementary material for: Effects of In-Person Navigation to Address Family Social Needs on Child Health Care Utilization: A Randomized Clinical Trial
Source: JAMA Netw Open. 2020 Jun 1;3(6):e206445. doi: 10.1001/jamanetworkopen.2020.6445 (PMC7265099; doi:10.1001/jamanetworkopen.2020.6445)
Supplement: Supplement 2. — eTable 1. Characteristics of the Sample Excluded vs the Sample Analyzed eTable 2. Risk of Health Care Utilization Outcome of Navigator Intervention Group Compared With Active Control Group 4 Months After Enrollment eTable 3. Risk of Health Care Utilization Outcome of Navigator Intervention Group Compared With Active Control Group Using Multiple Imputation [file jamanetwopen-3-e206445-s002.pdf]

## Supplementary Online Content

Pantell MS, Hessler D, Long D, et al. Effects of in-person navigation to address family social needs on child health care utilization: a randomized clinical trial. JAMA Netw Open. 2020;3(6):e206445. doi:10.1001/jamanetworkopen.2020.6445

**eTable 1.** Characteristics of the Sample Excluded vs the Sample Analyzed
**eTable 2.** Risk of Healthcare Utilization Outcome of Navigator Intervention Group Compared With Active Control Group 4 Months After Enrollment
**eTable 3.** Risk of Healthcare Utilization Outcome of Navigator Intervention Group Compared With Active Control Group Using Multiple Imputation

This supplementary material has been provided by the authors to give readers additional information about their work.

© 2020 Pantell MS et al. *JAMA Network Open*.

**eTable 1. Characteristics of the Sample Excluded vs the Sample Analyzed**

| N (%), unless otherwise noted[a] | Sample<br>Excluded<br>N = 509 | <br>Analyzed<br>N = 1,300 |
|---|---|---|
| Intervention arm | 274 (53.8) | 663 (51.0) |
| Site | | |
| UCSF Benioff Children's Hospital Oakland | 239 (47.0) | 649 (49.9) |
| Zuckerberg San Francisco General Hospital and Trauma Center | 270 (53.1) | 651 (50.1) |
| Setting | | |
| Urgent care | 397 (78.0) | 840 (64.6) |
| Primary care | 112 (22.0) | 460 (35.4) |
| Child age | | |
| 0-5 | 291/459 (63.4) | 779 (59.9) |
| 6-12 | 120/459 (61.2) | 390 (30.0) |
| 13-18 | 48/459 (10.5) | 131 (10.1) |
| Child sex female | 261/497 (52.5) | 652 (50.2) |
| Race/Ethnicity | | |
| Hispanic | 308/506 (60.9) | 723 (55.6) |
| Non-Hispanic Black | 115/506 (22.7) | 358 (27.5) |
| Asian | 20/506 (4.0) | 66 (5.1) |
| Non-Hispanic White | 20/506 (4.0) | 54 (4.2) |
| Other race/ethnicity | 42/506 (8.5) | 99 (7.6) |
| Caregiver language | | |
| Spanish | 225/509 (44.2) | 422 (32.5) |
| English | 284/509 (55.8) | 878 (67.5) |
| Caregiver age, N/mean (SD), y | | |
| Caregiver sex female | 407/470 (86.6) | 1,127 (86.7) |
| Caregiver relationship to child | | |
| Parent | 454/473 (96.0) | 1,254 (96.6) |
| Legal foster parent or guardian | 1/473 (0.2) | 4 (0.3) |
| Other adult family member | 18/473 (3.8) | 40 (3.1) |
| Caregiver education | | |
| Less than 8th grade | 91/451 (20.2) | 216 (16.8) |
| Some high school | 78/451 (17.3) | 225 (17.5) |
| High school graduate or GED | 118/451 (26.2) | 342 (26.6) |
| Some college | 104/451 (23.1) | 217 (24.6) |
| College graduate | 60/451 (13.3) | 188 (14.6) |
| No. of social needs, mean (SD) | 2.70 (2.3) | 2.76 (2.1) |
| Baseline health status | | |
| Fair/poor | 30/506 (5.9) | 118 (9.1) |
| Good | 178/506 (35.2) | 382 (29.4) |
| Very good | 132/506 (26.1) | 338 (26.0) |
| Excellent | 166/506 (32.8) | 462 (35.5) |
| Asked about nonmedical needs in past year | 100/493 (20.3) | 219 (16.9) |
| Below federal poverty level | 295/391 (75.5) | 820/1,116 (73.5) |

[a]Denominator is out of sample N unless otherwise noted.

GED = general equivalency diploma

© 2020 Pantell MS et al. *JAMA Network Open*.

**eTable 2. Risk of Healthcare Utilization Outcome of Navigator Intervention Group Compared With Active Control Group 4 Months After Enrollment**

| | N = 1,809 Model 1 | | N = 1,809 Model 2 | | N = 1,809 Model 3 | |
|---|---|---|---|---|---|---|
| | HR (95% CI) | *P*-Value[a] | HR (95% CI) | *P*-Value | HR (95% CI) | *P*-Value[a] |
| ED Visit w/in 4 Months | 0.89 (0.69-1.15) | 0.39 | 0.88 (0.68-1.14) | 0.33 | 0.91 (0.68-1.21) | 0.50 |
| Hospitalized w/in 4 Months | 0.44 (0.22-0.87) | 0.02 | 0.43 (0.22-0.86) | 0.02 | 0.40 (0.18-0.89) | 0.03 |

ED = emergency department; HR = hazard ratio; CI = confidence interval

Model 1: Unadjusted

number of social risks, clinical site, clinic setting, and being asked about nonmedical needs in the past twelve months in a clinical setting.

Model 3: Additionally adjusted for poverty.

© 2020 Pantell MS et al. *JAMA Network Open*.

**eTable 3. Risk of Healthcare Utilization Outcome of Navigator Intervention Group Compared With Active Control Group Using Multiple Imputation**

| | N = 1,300 | | N = 1,300 | | N = 1,116 | |
|---|---|---|---|---|---|---|
| | Model 1 | | Model 2 | | Model 3 | |
| | HR (95% CI) | *P*-Value[a] | HR (95% CI) | *P*-Value | HR (95% CI) | *P*-Value[a] |
| ED Visit w/in 1 Yr | 0.91 (0.78-1.96) | 0.21 | 0.90 (0.77-1.05) | 0.19 | 0.90 (0.77-1.05) | 0.17 |
| Hospitalized w/in 1 Yr | 0.61 (0.39-0.97) | 0.04 | 0.60 (0.38-0.96) | 0.03 | 0.61 (0.38-0.96) | 0.03 |

ED = emergency department; HR = hazard ratio; CI = confidence interval

Model 1: Unadjusted

number of social risks, clinical site, clinic setting, and being asked about nonmedical needs in the past twelve months in a clinical setting.

Model 3: Additionally adjusted for poverty.

[a] *P*-values rounded to two decimals.

© 2020 Pantell MS et al. *JAMA Network Open*.
